# Artificial intelligence meets genomic selection: comparing deep learning and GBLUP across diverse plant datasets

**DOI:** 10.3389/fgene.2025.1568705

**Published:** 2025-04-29

**Authors:** Abelardo Montesinos-López, Osval A. Montesinos-López, Sofia Ramos-Pulido, Brandon Alejandro Mosqueda-González, Edgar Alejandro Guerrero-Arroyo, José Crossa, Rodomiro Ortiz

**Affiliations:** ^1^ Departamento de Matemáticas, Centro Universitario de Ciencias Exactas e Ingenierías (CUCEI), Universidad de Guadalajara, Guadalajara, Jalisco, Mexico; ^2^ Facultad de Telemática, Universidad de Colima, Colima, Mexico; ^3^ Institut National des Sciences Appliquées de Lyon: Lyon, Villeurbanne, France; ^4^ International Maize and Wheat Improvement Center (CIMMYT), Texcoco, Mexico; ^5^ Colegio de Postgraduate Program in Socioeconomics, Statistics, and Informatics (PSEI) at the Montecillo Campus, Texcoco, Mexico; ^6^ Department of Plant Breeding at SLU, Swedish University of Agricultural Sciences, Uppsala, Sweden

**Keywords:** benchmarking, deep learning, GBLUP, genomic selection, plant breeding

## Abstract

To enhance the implementation of genomic selection (GS) in plant breeding, we conducted a comprehensive comparative analysis of deep learning (DL) models and genomic best linear unbiased predictor (GBLUP) methods across 14 real-world datasets derived from diverse plant breeding programs. We evaluated model performance by meticulously tuning hyperparameters specific to each dataset, aiming to maximize predictive accuracy and reliability. Our results demonstrated that DL models effectively captured complex, non-linear genetic patterns, frequently providing superior predictive performance compared to GBLUP, especially in smaller datasets. However, neither method consistently outperformed the other across all evaluated traits and scenarios. The analysis revealed that the success of DL models significantly depended on careful parameter optimization, reinforcing the importance of rigorous model tuning procedures. In the discussion, we emphasize the complementary nature of DL and GBLUP methods, highlighting that the choice between these models should be driven by the specific characteristics of the traits under study and the evaluation metrics prioritized in breeding programs. These insights contribute practical guidelines for selecting and optimizing genomic prediction models to achieve robust outcomes in plant breeding contexts.

## Introduction

Genomic selection (GS) has emerged as a transformative tool in contemporary breeding programs, leveraging genomic data to predict the genetic potential and performance of individuals. By using dense marker information across the genome, this approach enables the selection of candidates with desirable traits more efficiently than traditional breeding methods. Unlike conventional approaches that rely heavily on extensive and resource-intensive phenotypic evaluations, genomic selection accelerates the breeding process by identifying superior individuals early, thereby saving time and resources. This methodology has fundamentally reshaped both animal and plant breeding, facilitating the development of high-performing varieties and breeds tailored to meet the challenges of food security and climate resilience (Meuwissen et al., 2001; Tester and Langridge, 2010; Varshney et al., 2021; Hickey et al., 2017).

In this context, employing advanced computational tools to improve the accuracy of predictions is vital for enhancing the efficiency of breeding programs and minimizing the time and resources needed for developing new varieties. Among these tools, Genomic Best Linear Unbiased Prediction (GBLUP) has been a benchmark for genomic prediction due to its reliability, scalability, and ease of interpretation (VanRaden, 2008; de los Campos and Gianola, 2023). GBLUP uses genomic markers within linear mixed models to produce accurate estimates of genetic values, especially for traits predominantly influenced by additive genetic effects. Its computational efficiency and strong statistical framework have established it as a cornerstone in both animal and plant breeding applications. However, as traits exhibit increasing complexity, including non-linear interactions and genotype-by-environment effects, the constraints of linear models become evident, highlighting the need to adopt more versatile and adaptive methodologies (Meuwissen et al., 2001; Crossa et al., 2017).

Deep Learning (DL) techniques have established themselves as a dynamic and robust alternative in genomic prediction, excelling in the modeling of intricate, non-linear interactions among genomic markers (LeCun et al., 2015). By leveraging their capacity to capture epistatic interactions, accommodate heterogeneous data types, and effectively process high-dimensional datasets, DL models demonstrate substantial promise in genomic prediction tasks (Montesinos-López et al., 2018; Ma et al., 2018; Montesinos-López et al., 2023). Notably, DL methodologies have been successfully applied to forecast traits across agriculture and animal science, including agronomic traits like drought tolerance, disease resistance, and crop yield, as well as animal production traits such as milk production in Jersey cows. These approaches frequently outperform traditional methods, showcasing their versatility and predictive power in complex biological systems occasionally/often outperforming traditional approaches (Gianola et al., 2011; Upadhyaya et al., 2024; Montesinos-López et al., 2024a). These strengths position DL as an invaluable tool for plant breeders striving to enhance predictive accuracy for traits governed by complex genetic architectures (Zingaretti et al., 2020).

Recent studies comparing DL and GBLUP emphasize the trade-offs between these approaches. GBLUP remains a reliable method for traits with predominantly additive genetic effects and large reference populations due to its simplicity and interpretability (VanRaden, 2008). On the other hand, DL is particularly advantageous for modeling non-linear and epistatic interactions, making it well-suited for complex traits and datasets that incorporate diverse genomic, environmental, and phenotypic information (Azodi et al., 2019; Zingaretti et al., 2020). For instance, Pérez-Enciso and Zingaretti (2019) demonstrated that DL models outperformed traditional approaches in predicting complex traits across varying environments, highlighting DL’s potential for advancing modern breeding strategies.

There are several reasons why to compare genomic prediction accuracy of DL with GBLUP. GBLUP is useful in genomic prediction. Comparing both helps identify scenarios where DL offers improvements or underperforms. Unlike typical big data applications, this study investigates whether DL can provide meaningful advantages in genomic prediction with smaller datasets. Furthermore, note that GBLUP assumes linear relationships, while DL can model complex interactions. This comparison tests the relevance of DL’s flexibility for traits with non-linear genetic architectures. Comparing DL with GBLUP helps validate if DL innovations justify their added complexity in genomic prediction tasks, especially in data-limited contexts (Gianola and Rosa, 2015; Crossa et al., 2017; Montesinos-López et al., 2021).

This study aims to provide a comprehensive comparison between GBLUP and multilayer perceptron’s (MLPs), one of the most applied DL architectures for genomic prediction. MLPs, referred to hereafter as DL, are also known as feedforward neural networks and have proven effective in predicting complex phenotypes based on genomic data (Montesinos-López et al., 2021; Farooq et al., 2024). Specifically, it evaluates their performance across different genetic architectures, population sizes, and levels of marker density. By contrasting the linear framework of GBLUP compared with the non-linear capabilities of DL seeks to elucidate the trade-offs and complementarities of these methods, ultimately guiding the selection of appropriate tools for genomic prediction.

The following sections present the dataset used and its main characteristics, describe the models and assumptions underlying the GBLUP and DL models, and outline the experimental design, including the datasets and evaluation metrics employed to assess the performance of these methods. Finally, we discuss the implications of our findings, providing guidance for researchers and practitioners in selecting optimal strategies for genomic prediction.

This study stands out by comparing DL and GBLUP across 14 datasets representing diverse crops, traits, and sample sizes. Unlike many previous studies focused on large or single-species datasets, our work evaluates model performance under a broader range of conditions, including smaller datasets. This provides a more realistic benchmark for breeding programs, especially those with limited resources or working on less-studied crops.

## Materials and methods

### Datasets

The dataset used consists of the 14 datasets described in the study by Montesinos-López et al. (2024b), which correspond to the BLUEs of line effects obtained by removing the environment effect and the design effect (either a randomized complete block design (RCBD) or an alpha lattice experimental design, depending on the dataset). The number of lines, markers and traits measures in each dataset are given in Table 1.

**TABLE 1:** Number of lines, markers and traits for each data set.

Data	No. of lines	No. of markers	Traits
Disease	438	11,617	PTR, SB, SN
EYT_1	776	2,038	DTHD, DTMT, GY, Height
EYT_2	775	2,038	DTHD, DTMT, GY, Height
EYT_3	964	2,038	DTHD, DTMT, GY, Height
Groundnut	318	8,268	NPP, PYPP, SYPP, YPH
Indica	327	16,383	GC, GY, PH, PHR
Japonica	320	16,383	GC, GY, PH, PHR
Maize	722	54,113	Y
Wheat_1	1,301	78,606	GY
Wheat_2	1,403	78,606	GY
Wheat_3	1,403	78,606	GY
Wheat_4	1,388	78,606	GY
Wheat_5	1,398	78,606	GY
Wheat_6	1,277	78,606	GY

Traits are: *Pyrenophora tritici-repentis* (PTR), spot blocht (SB), *and Septoria nodorum* (SN), days to heading (DTHD), days to maturity (DTMT), Grain Yield (GY, Y), plant height (HEIGHT), Number of Pods per Plant (NPP), Pod Yield per Plant (PYPP), Seed Yield per Plant (SYPP), Yield per Hectare (YPH), Gel Consistency (GC), Grain yield (GY), Plant height (PH), Plant Height Reduction (PHR).

While the wheat datasets are described in detail in Montesinos-López et al. (2024b), the remaining datasets—such as Groundnut, Indica, Japonica, Maize, and the multi-trait Disease dataset—are sourced from real breeding programs and have been used in previous studies (e.g., Montesinos-López et al., 2018; Montesinos-López et al., 2021). Briefly, these datasets represent a variety of crops and traits relevant to grain yield, disease resistance, and agronomic performance, all preprocessed into BLUEs to remove environmental and design effects.

The complexity of traits was qualitatively assessed based on known biological and genetic characteristics, rather than heritability estimates alone. Traits such as grain yield (GY), disease resistance (PTR, SB, SN), and plant height reduction (PHR) are classified as complex due to their polygenic architecture, sensitivity to environmental interactions, and involvement of epistasis or non-additive effects. In contrast, traits like gel consistency (GC), days to heading (DTHD), and plant height (PH) are considered simpler because they are primarily governed by additive effects and exhibit more stable inheritance across environments.

The 14 datasets span a range of crops and traits with varying complexity. Trait architectures include both simple (e.g., plant height, gel consistency) and complex traits (e.g., grain yield, disease resistance), influenced by polygenic effects and genotype-by-environment (G × E) interactions. The datasets also vary in sample size, from 318 (Groundnut) to 1,403 lines (Wheat_2), and in marker density, from 2,038 SNPs (EYT datasets) to over 78,000 SNPs (Wheat datasets). This diversity provides a realistic setting to evaluate model robustness across different genomic prediction challenges.

## Models

### DL model

For a univariate response $Y_{i}$ , the multilayer perceptron deep learning (MLPDL) model (Goodfellow et al., 2016) with L hidden layers, $N_{l}$ units in layer l,l=1,…,L , and a linear activation in the output layer, in vector-matrix representation, is given by

$$Y_{i}=w_{0}^{\left(0\right)}+W_{1}^{\left(0\right)}x_{i}^{\left(L\right)}+\epsilon_{i}$$ (1)

where $x_{i}^{\left(l\right)}=g_{l}\left(w_{0}^{\left(l\right)}+W_{1}^{\left(l\right)}x_{i}^{\left(l-1\right)}\right)$ for l=1,…,L , with $x_{i}^{\left(0\right)}=x_{i},$ the covariable p×1 vector of individual i in the sample; $w_{0}^{\left(l\right)}$ , $W_{1}^{\left(l\right)}$ , l=1,…,L , represent the bias vector of size $N_{l}\times 1$ and the weight matrix of size $N_{l}\times N_{l-1}$ for hidden layers, with $N_{0}=p$ . Similarly in Equation 1, $w_{0}^{\left(0\right)}$ and $W_{1}^{\left(0\right)}$ are the bias and the weight vector ( $1\times N_{L}$ ) for the output layer; $g_{l}$ denotes the activation function for layer l , applied element-wise to an input vector. In this case, the Rectified Linear Unit (ReLU) activation function was used; $\epsilon_{i},i=1,\ldots ,n$ , are the errors terms assumed to be independent with normal distribution with mean 0 and common variance.

The complete model architecture was based on ResNet (Residual Network) with residual connections added to mitigate the vanishing gradient problem (He et al., 2016). The ResNet employed consisted of two sequential layers. A batch normalization layer was added after each dense layer and before the activation function to standardize the outputs, keeping the mean near 0 and the standard deviation close to 1. Batch normalization was applied after each hidden layer to stabilize training. To prevent overfitting while maintaining output flexibility, L2 regularization was enforced on all weights, excluding the bias of the output layer. Lastly, a mean squared error loss function was employed, to which the L2 penalty was added, scaled by a regularization parameter ( λ ). This parameter controls the degree of weight shrinkage, reducing model complexity and mitigating overfitting. Thus, the parameters (bias and weights) of model (1) were estimated using the induced log-likelihood of the error distribution, penalized by an L2 quadratic term, excluding the bias of the output layer $\left(w_{0}^{\left(0\right)}\right)$ .

In subsequent mentions, DL will refer to this completely described deep learning model (1).

The model was implemented with the Torch library in Python (Paszke et al., 2019), with a Batch_size value equal to the training size to take advantage of GPU. The model was trained for 128 epochs, a value chosen to ensure sufficient learning without overfitting, balancing model performance and computational efficiency. To optimize the weights of the deep learning model, the Adam optimizer—the most popular and widely optimizer used in deep learning—was adopted. Additionally, a learning rate scheduler (StepLR) was used to systematically update the initial learning rate ( $l_{r}$ ) by multiplying it by a specified factor $\left(\gamma \right)$ every 10 epochs. This gradual reduction in learning rate prevents premature convergence to suboptimal solutions and facilitates finer adjustments in later training stages.

To simplify the search space and training process, we specified the units only for the first hidden layer, $N_{1}$ , while the units in the subsequent layers were set as the largest integer less than half of the units in the preceding layer, that is, $N_{l}=\lfloor N_{l-1}/\;2\rfloor$ for l=2,…,L .

The Hyperparameters tuned in the DL model (1) were the number of hidden layers ( L ), the number of units in first layer $N_{1},$ the regularization parameter ( λ ), the initial learning rate ( $l_{r}$ ) and the factor $\left(\gamma \right.$ ) in the StepLR learning rate scheduler. These hyperparameters significantly impact model generalization and convergence behavior. They were optimized using the bayes_opt library (Gardner et al., 2014; Nogueira, 2014) over 250 iterations, employing Bayesian Optimization, a strategy well-suited for non-convex and multimodal optimization problems that efficiently balances exploration and exploitation (Shahriari et al., 2015). The tuning process aimed to minimize the average mean squared error on the validation set, using an inner 10-fold cross-validation strategy to ensure robust model selection and reduce overfitting risk. Table 2 lists the hyperparameters along with their explored search spaces, reflecting the range of values considered during the optimization process.

**TABLE 2:** Hyperparameter values tuned in the DL model (1) and their search space.

Hyperparameter	Search space
Number of hidden layers ( L )	$\left[1,4\right]$
Number of units in the first layer ( $N_{1}$ )	$\left[64,512\right]$
Regularization parameter in log-scale ( $l\lambda =\log\left(\lambda \right)$ )	$\left[-10,-1\right]$
Initial learning rate in log-scale ( ${ll}_{r}=\log\left(l_{r}\right)$ )	$\left[-10,0\right]$
Factor γ in the StepLR learning rate scheduler in log-scale ( $l\gamma =\log\left(\gamma \right)$ )	$\left[\log\left(0.05\right),\log\left(0.95\right)\right]$

The models were executed on a computer with 128 GB of RAM and 40 cores, together with a 48 GB GPU, and the experiments were conducted using Python version 3.9.19 and torch 2.4.0. Across all evaluations performed for each trait and each dataset, the total training time ranged between 9 and 18 h, resulting in approximately 420 h of training to process all evaluations for all traits.

The choice of the Adam optimizer was based on its proven stability and popularity in deep learning applications, especially for non-convex problems like genomic prediction. As noted in our earlier review (Montesinos-López et al., 2021), Adam is widely adopted due to its adaptive learning rates and efficiency in handling sparse gradients. We adopted it here for its robustness and ease of implementation in high-dimensional genomic datasets. Hyperparameter settings (number of layers, units, regularization, learning rate) were optimized using Bayesian optimization across 250 iterations, with ranges informed by prior DL studies in genomics (e.g., Montesinos-López et al., 2023). These ranges were selected to ensure both sufficient model complexity and regularization control to prevent overfitting in smaller datasets.

To ensure full reproducibility, all source code, and model configurations have been made available in a public GitHub repository: https://github.com/GHAML1/AI-DL-and-GBLUP-06Abr25.

### GBLUP model

The GBLUP model is defined as

$$Y_{j}=\mu +g_{j}+\epsilon_{j}$$

where the genotypic effects $g={\left(g_{1},\ldots ,g_{J}\right)}^{T}$ are jointly distributed as multivariate normal distribution with vector means zero, 0 , and variance-covariance $\sigma_{g}^{2}G$ , with G the genomic relationship matrix as computed by Meuwissen et al. (2001). Furthermore, the error terms ( $\epsilon_{j}$ ; j=1,…,J ) in the model are assumed to be independent random normal variables with mean 0 and variance $\sigma^{2}$ .

A Bayesian estimation of this model was conducted, assuming the following independent prior distributions for the model parameters

$$f\left(\mu ,\sigma_{g}^{2},\sigma^{2}\right)\propto f\left(\sigma_{g}^{2}\right)f\left(\sigma^{2}\right)$$

where $f\left(\sigma_{g}^{2}\right)$ and $f\left(\sigma^{2}\right)$ denote the scaled-inverse chi-squared distribution with parameters $\left(v_{g},S_{g}\right)$ and $\left(v,S\right)$ , respectively. The model was implemented using the BGLR package under the “RKHS” model with default hyperparameters for the priors.

## Evaluation of predictive accuracy

To evaluate the performance prediction of model (1) and compare it with the GBLUP model (2), a 10-fold-cross-validation (10FCV) strategy was adopted by using the marker information (X) available in each of the 14 datasets as predictors, containing between one to four measured traits. Specifically, one dataset (Disease) has three traits, six datasets have four traits (EYT_1-EYT_3, Groundnut, Indica, and Japonica), and seven datasets have only one trait (Maize and Wheat_1-Wheat_6); see Table 1. For evaluation of the prediction accuracy, we adopted the following metrics: Pearson’s correlation (Cor), normalized root mean squared error (NRMSE), and percentage of matching in top 20% (Matching20). The last metric, Matching20, represents the percentage of lines ranked within the top 20% that are correctly predicted to fall within this top 20%.

### Data availability

The 14 phenotypic and genotypic data sets used in this study are available at the following link: https://github.com/osval78/Refaning_Penalized_Regression.

## Results

In this section, we present the summary results of the ten-fold cross-validation strategy 10FCV evaluation performance for the DL model (1) and the GBLUP model (2) across the 14 datasets and traits listed in Table 1. All the results are displayed in Supplementary Appendix Table A1, where the first column of this identifies the model (DL or GBLUP) evaluated on the dataset and trait specified in the second and third columns, respectively. The final three columns report the mean and standard deviation of the evaluated metrics (Cor (SD), NRMSE (SD), and Matching20 (SD)) computed over the 10-fold cross-validation (10FCV). The results of Supplementary Appendix Table A1 are first presented for each dataset and then across datasets.

### Disease dataset

**FIGURE 1:**
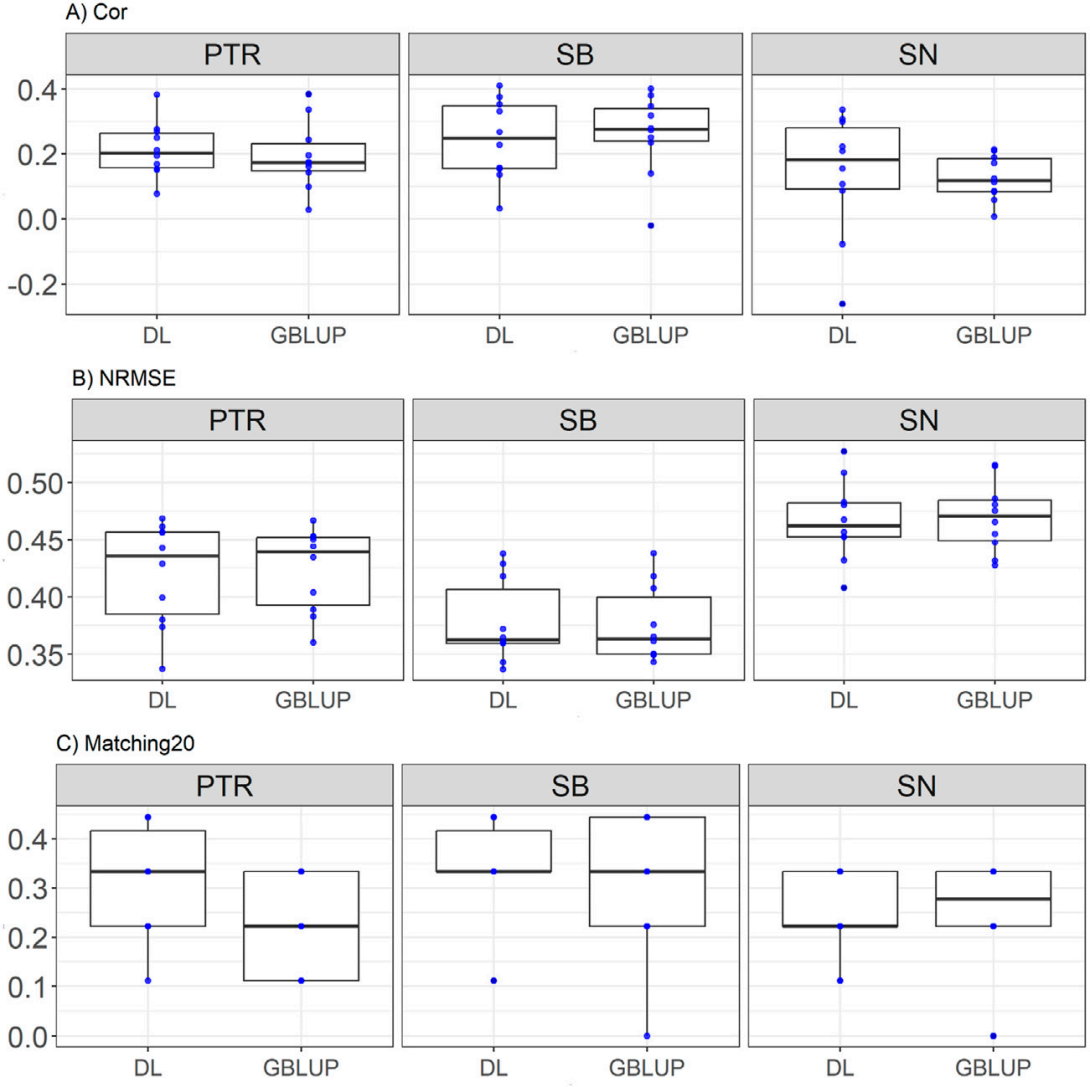
Box plots depicting the performance of DL and GBLUP models across ten-fold cross-validation for Disease data in each trait, *Pyrenophora tritici-repentis* (PTR), spot blocht (SB), and *Septoria nodorum* (SN). **(A)** Box plot of the Pearson’s correlation (Cor) between observed and predicted values for each of the three traits across ten-fold cross-validation. **(B)** Box plot of the normalized root mean square error (NRMSE) between observed and predicted values for each trait. **(C)** Box plot of the top 20% matching percentages (Matching20) for observed and predicted values.

This dataset included traits *Pyrenophora tritici-repentis* (PTR), spot blocht (SB), and *Septoria nodorum* (SN), representing complex disease-related features. Here it is observed that for PTR, DL outperforms GBLUP in all metrics: it shows a higher correlation (Cor = 0.213 vs. 0.193, with a relative improvement of 10.15%), a lower normalized error (NRMSE = 0.42 vs. 0.423, relative improvement of 0.75%), and superior predictive ability in the top 20 matches (Matching20 = 0.311 vs. 0.222, relative improvement of 40%). For SB, the results are more balanced, with GBLUP showing a slightly higher correlation (Cor = 0.26 vs. 0.244, relative improvement of 6.49%) and a lower normalized error (NRMSE = 0.375 vs. 0.378, relative improvement of 0.59%), while DL stands out in Matching20 (0.344 vs. 0.311, relative improvement of 10.71%). Finally, for SN, DL exhibits marginally better correlation (Cor = 0.138 vs. 0.125, relative improvement of 10.55%) and a lower normalized error (NRMSE = 0.466 vs. 0.469, relative improvement of 0.66%), while GBLUP shows a slight advantage in Matching20 (0.255 vs. 0.244, relative improvement of 4.54%). These results are displayed in Figure 1.

### EYT_1 dataset

**FIGURE 2:**
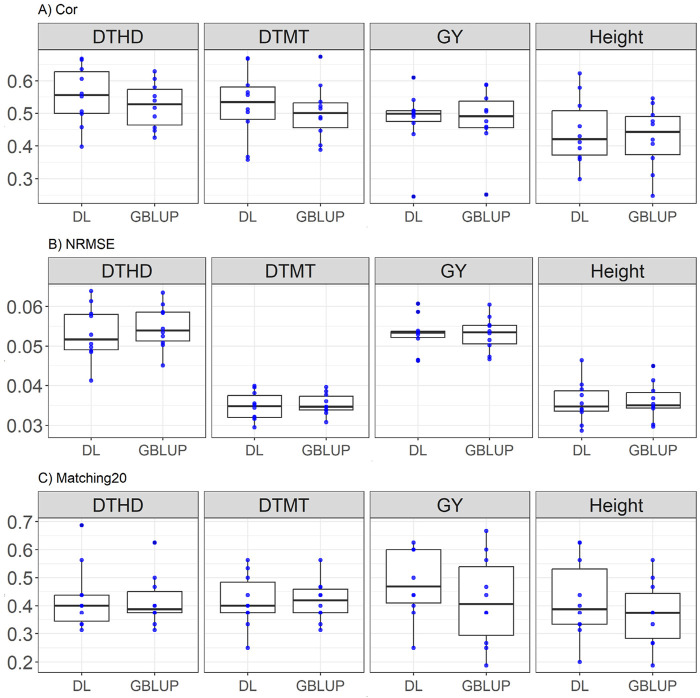
Box plots depicting the performance of DL and GBLUP models across ten-fold cross-validation for EYT_1 data in each trait, days to heading (DTHD), days to maturity (DTMT), grain yield (GY) and plant height (Height). **(A)** Box plot of the Pearson’s correlation (Cor) between observed and predicted values for each of the three traits across ten-fold cross-validation. **(B)** Box plot of the normalized root mean square error (NRMSE) between observed and predicted values for each trait. **(C)** Box plot of the top 20% matching percentages (Matching20) for observed and predicted values.

Here the comparison between DL and GBLUP models across traits reveals that DL generally outperforms GBLUP in most metrics. For days to heading (DTHD), DL shows higher correlation (0.554 vs. 0.523) with a relative improvement of 5.81%, lower NRMSE (0.053 vs. 0.054, a 2.72% improvement), and slightly better Matching20 (0.427 vs. 0.416, a 2.80% improvement). Similarly, in days to maturity (DTMT), DL demonstrates superior correlation (0.525 vs. 0.503, a 4.36% improvement), lower NRMSE (0.034 vs. 0.035, a 1.43% improvement), and equivalent Matching20 (0.416 for both). For GY, GBLUP marginally outperforms DL in correlation (0.481 vs. 0.479, a 0.34% advantage), while DL excels in Matching20 (0.482 vs. 0.418, a 15.22% improvement) with equal NRMSE (0.053). In plant Height, DL surpasses GBLUP in correlation (0.444 vs. 0.425, a 4.29% improvement), NRMSE (0.035 vs. 0.036, a 0.53% improvement), and Matching20 (0.42 vs. 0.37, a 13.31% improvement). These results are displayed in Figure 2.

### EYT_2 dataset

**FIGURE 3:**
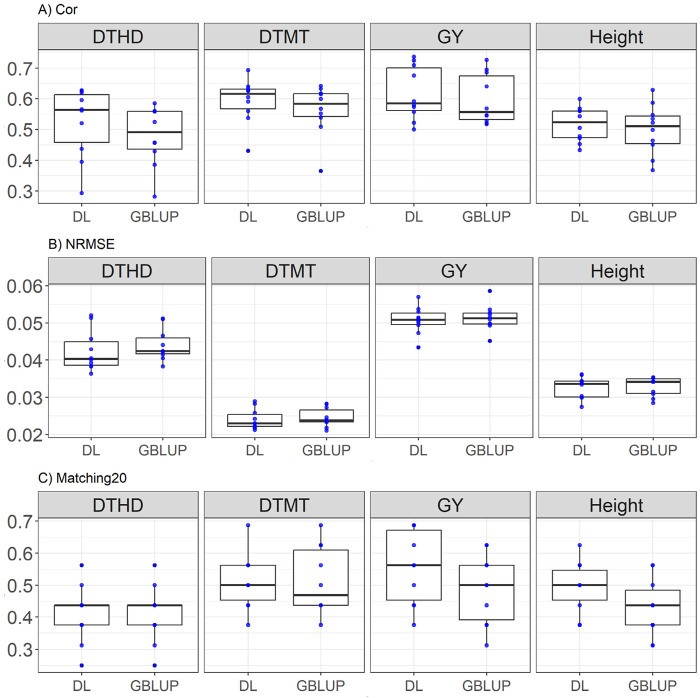
Box plots depicting the performance of DL and GBLUP models across ten-fold cross-validation for EYT_2 data in each trait, days to heading (DTHD), days to maturity (DTMT), grain yield (GY) and plant height (Height). **(A)** Box plot of the Pearson’s correlation (Cor) between observed and predicted values for each of the three traits across ten-fold cross-validation. **(B)** Box plot of the normalized root mean square error (NRMSE) between observed and predicted values for each trait. **(C)** Box plot of the top 20% matching percentages (Matching20) for observed and predicted values.

DL generally outperforms GBLUP across most metrics. For DTHD, DL achieves a higher correlation (0.523 vs. 0.479), showing an improvement of 9.23%, and a lower NRMSE (0.042 vs. 0.044, a 3.4% improvement), while both models have equal Matching20 (0.412). For DTMT, DL surpasses GBLUP with a higher correlation (0.593 vs. 0.563, a 5.37% improvement) and equal NRMSE (0.024), with identical Matching20 (0.512). In GY, DL demonstrates a higher correlation (0.616 vs. 0.597, a 3.29% improvement) and lower NRMSE (0.05 vs. 0.051, a 1.28% improvement), while significantly outperforming Matching20 (0.556 vs. 0.487, a 14.10% improvement). For Height, DL achieves a better correlation (0.516 vs. 0.499, a 3.48% improvement) and substantially outperforms GBLUP in Matching20 (0.493 vs. 0.431, a 14.10% improvement), while both models show equal NRMSE (0.032). These results are shown in Figure 3.

### EYT_3 dataset

**FIGURE 4:**
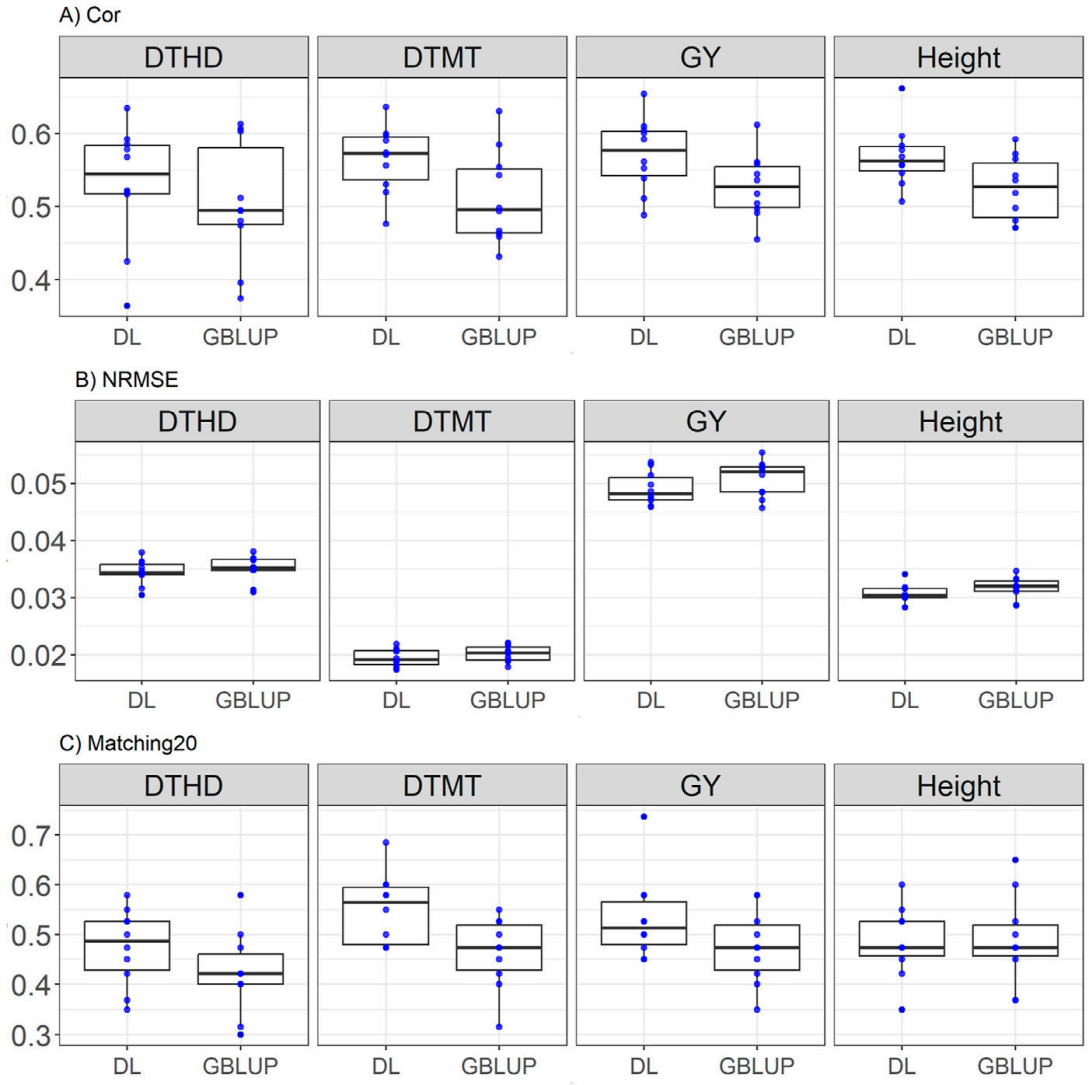
Box plots depicting the performance of DL and GBLUP models across ten-fold cross-validation for EYT_3 data in each trait, days to heading (DTHD), days to maturity (DTMT), grain yield (GY) and plant height (Height). **(A)** Box plot of the Pearson’s correlation (Cor) between observed and predicted values for each of the three traits across ten-fold cross-validation. **(B)** Box plot of the normalized root mean square error (NRMSE) between observed and predicted values for each trait. **(C)** Box plot of the top 20% matching percentages (Matching20) for observed and predicted values.

Here the results are consistent with the trends observed in EYT_2, with DL exhibiting slightly higher correlation and Matching20 in most traits. DL outperformed GBLUP across most traits, with notable improvements in Matching20 for DTMT (0.564 vs. 512) and GY (0.571 vs. 0.527). Correlation improvements were moderate but consistent for DL, while NRMSE differences remained minimal. For DTHD, DL achieves a higher correlation (0.53 vs. 0.504), representing an improvement of 5.04%, and a lower NRMSE (0.034 vs. 0.035), corresponding to a 1.7% reduction. Additionally, in Matching20, DL outperformed GBLUP by 12.12% (0.474 vs. 0.423). For DTMT, DL surpasses GBLUP with a higher correlation (0.564 vs. 0.512, a 10.28% improvement) and an almost identical NRMSE (0.019 vs. 0.020), with large difference between Matching20 (0.551 vs. 0.463, 18.89% improvement). In GY, DL demonstrates a higher correlation (0.571 vs. 0.527, a 8.28% improvement) and lower NRMSE (0.049 vs. 0.050, a 3.39% improvement), while substantially outperforms GBLUP in Matching20 (0.532 vs. 0.475, a 11.96% improvement). For Height, DL achieves a better correlation (0.568 vs. 0.524, an 8.35% improvement) and a slight decrease in Matching20 (0.484 vs. 0.488, a 0.81% reduction), while both models show similar NRMSE (0.03 vs. 0.031). The results are presented in Figure 4.

### Groundnut dataset

Traits included are: Number of Pods per Plant (NPP), Pod Yield per Plant (PYPP), Seed Yield per Plant (SYPP), Yield per Hectare (YPH).

**FIGURE 5:**
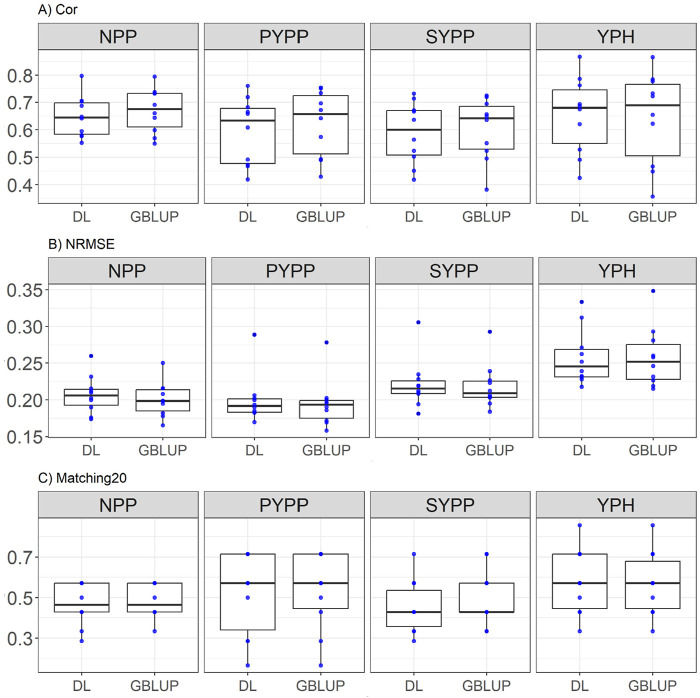
Box plots depicting the performance of DL and GBLUP models across ten-fold cross-validation for *Groundnut* data in each trait, Number of Pods per Plant (NPP), Pod Yield per Plant (PYPP), Seed Yield per Plant (SYPP), Yield per Hectare (YPH). **(A)** Box plot of the Pearson’s correlation (Cor) between observed and predicted values for each of the three traits across ten-fold cross-validation. **(B)** Box plot of the normalized root mean square error (NRMSE) between observed and predicted values for each trait. **(C)** Box plot of the top 20% matching percentages (Matching20) for observed and predicted values.

NPP: GBLUP marginally outperforms DL in correlation (0.67 vs. 0.648, a 3.46% advantage) and in NRMSE (0.206 vs. 0.20 a 3.18% reduction), but only is slightly better in Matching20 (0.469 vs. 0.483, a 3.04% improvement). Details in Figure 5.

PYPP: GBLUP shows better correlation (0.623 vs. 0.594, a 4.9% improvement) and NRMSE (0.194 vs. 0.20, a 2.63% reduction), but only show a slightly better performance in Matching20 (0.538 vs. 0.523, a 2.72% advantage). Details in Figure 5.

SYPP: GBLUP edges DL in correlation (0.603 vs. 0.587, a 2.61% improvement), in NRMSE (0.218 vs. 0.221, a 1.2% reduction), and has an advantage in Matching20 (0.495 vs. 0.452, an improvement of 9.47%). Details in Figure 5.

YPH: DL outperforms GBLUP in correlation (0.653 vs. 0.643, a 1.59% improvement) and Matching20 (0.583 vs. 0.569, a 2.51% improvement), with equal NRMSE.

See Figure 5 for a visual representation of the results.

### Indica dataset

Traits included are, Gel Consistency (GC), Grain yield (GY), Plant height (PH), Plant Height Reduction (PHR).

GC: DL surpasses GBLUP in correlation (0.419 vs. 0.403, a 3.96% improvement) and Matching20 (0.442 for both), with marginally lower NRMSE (0.431 vs. 0.435, a 0.93% reduction). Details in Supplementary Appendix Figure B1.

GY: GBLUP marginally outperforms DL in correlation (0.628 vs. 0.616, a 1.95% improvement) and NRMSE (0.055 vs. 0.056, a 1.27% reduction), while DL lags in Matching20 (0.3 vs. 0.371, a 23.8% improvement for GBLUP). Details in Supplementary Appendix Figure B1.

PH: DL performs marginally better in correlation (0.541 vs. 0.537, a 0.79% improvement), while GBLUP has a slight edge in Matching20 (0.557 vs. 0.50, an 11.42% improvement). Both models show equal NRMSE. Details in Supplementary Appendix Figure B1.

PHR: GBLUP surpasses DL in correlation (0.431 vs. 0.378, a 13.91% improvement) and Matching20 (0.399 vs. 0.385, a 3.70% improvement), with slightly better NRMSE. Details in Supplementary Appendix Figure B1.

### Japonica dataset

Traits included are, Gel Consistency (GC), Grain yield (GY), Plant height (PH), Plant Height Reduction (PHR).

GC: GBLUP marginally outperforms DL in correlation (0.563 vs. 0.55, a 2.25% improvement) and in NRMSE (0.25 vs. 0.252, a 0.66% reduction). DL, however, excels in Matching20 (0.6 vs. 0.542, a 10.52% improvement). See details in Supplementary Appendix Figure B2.

GY: GBLUP shows higher correlation (0.571 vs. 0.505, a 12.95% improvement), better NRMSE (0.063 vs. 0.067, a 5.76% reduction) and better Matching20 (0.571 vs. 0.514, a 11.11% improvement). See details in Supplementary Appendix Figure B2.

PH: DL outperforms GBLUP in correlation (0.634 vs. 0.608, a 4.20% improvement) and GBLUP outperforms DL in Matching20 (0.485 vs. 0.5, a 3.00% improvement), with comparable NRMSE. See details in Supplementary Appendix Figure B2.

PHR: GBLUP has a slight advantage in correlation (0.545 vs. 0.536, a 1.68% improvement) and a moderate disadvantage in Matching20 (0.399 vs. 0.471, a 15.28% reduction), with equal NRMSE. See details in Supplementary Appendix Figure B2.

### Maize dataset

Here GBLUP outperforms DL in correlation (0.435 vs. 0.43, a 1.19% improvement) and Matching20 (0.446 vs. 0.433, a 3.07% improvement), with comparable NRMSE. See Supplementary Appendix Figure B3 for an overview of the results.

### Wheat datasets

In 5 out of the 6 Wheat datasets (grain yield, GY trait), DL outperforms GBLUP in terms of correlation, with improvements ranging from 1.36% to 8.93%, except for Wheat_3, where GBLUP slightly outperforms DL (0.478 vs. 0.475, a 0.80% improvement). Additionally, in Matching20, DL demonstrated better performance, with observed improvements of 2.83%, 0.88%, 5.55%, 7.25%, and 3.41% in the first five Wheat datasets. However, in the last dataset, GBLUP performed better than DL (0.55 vs. 0.519, a 5.92% improvement). For NRMSE, both models showed nearly identical performance, with a slight advantage for DL. Refer to Figure 4 for a detailed visualization of the results. See details in Supplementary Appendix Figure B4.

## Results across traits and datasets

**FIGURE 6:**
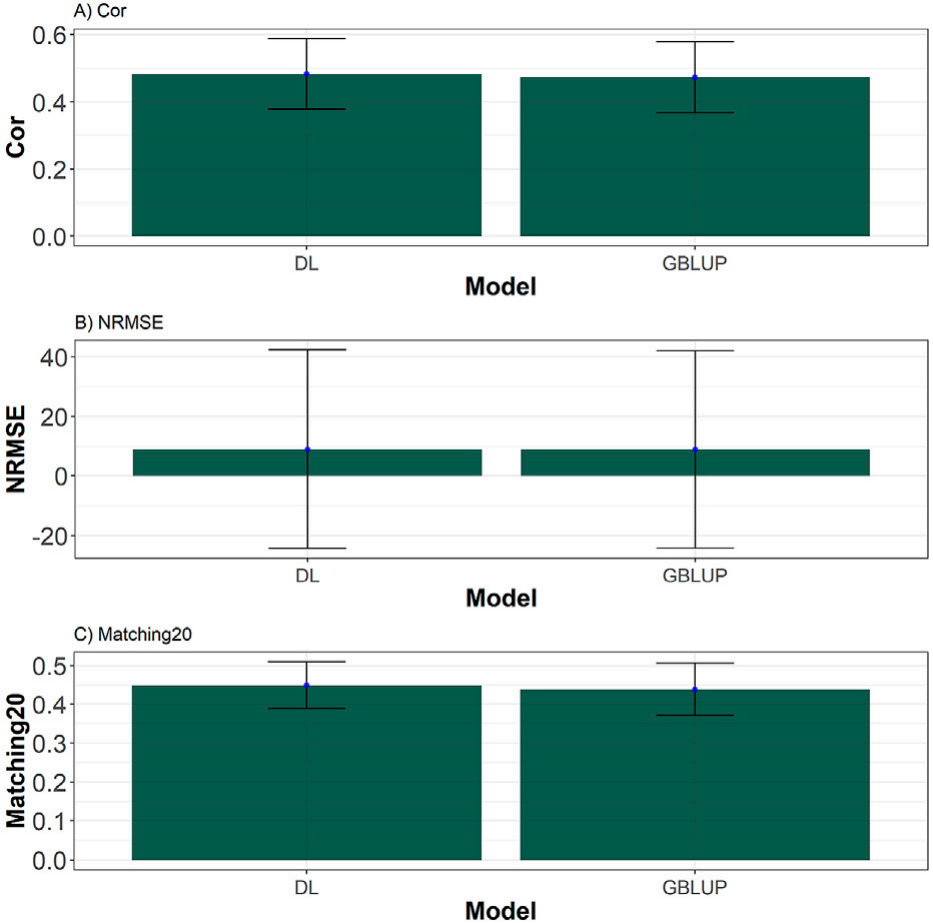
Barplots depicting the performance of DL and GBLUP models across ten-fold cross-validation across traits and data. **(A)** Barplot of the Pearson’s correlation (Cor) between observed and predicted values for each of the three traits across ten-fold cross-validation. **(B)** Barplot of the normalized root mean square error (NRMSE) between observed and predicted values for each trait. **(C)** Barplot of the top 20% matching percentages (Matching20) for observed and predicted values.

Then, by averaging the summary performance across data and traits of Supplementary Appendix Table A1 is computed (see Supplementary Appendix Table A2). From these summaries, we observe that, 10 out of the 14 datasets evaluated, the DL model demonstrated better performance in Cor, NRMSE, and Matching20. The comparison between Deep Learning (DL) and GBLUP models reveals distinct trends across the metrics of Pearson Correlation (Cor), Normalized Mean Squared Error (NRMSE), and Matching20. In terms of Cor, DL generally outperforms GBLUP in most datasets, such as EYT_1, EYT_2, EYT_3, Wheat_1, Wheat_2, Wheat_4, and Wheat_5, demonstrating better predictive accuracy in capturing the relationship between variables. However, GBLUP slightly surpasses DL in specific datasets like Groundnut, Indica, Japonica, and Maize, though these differences are minor and may not be statistically significant due to overlapping standard deviations. See Figure 6.

For NRMSE, the results between DL and GBLUP are nearly identical across all datasets, with minimal differences that fall within the margin of error. In datasets like Groundnut and Maize, GBLUP shows a slight advantage in terms of error minimization. However, these differences are not substantial enough to suggest a definitive edge for either model in this metric, indicating that both methods perform comparably in terms of prediction error. See Figure 6.

Matching20, which evaluates the ability to identify values in the top 20%, highlights a stronger performance for DL in datasets such as Disease, EYT_1, EYT_2, EYT_3, Wheat_1, Wheat_3, and Wheat_5. This suggests that DL is more effective at capturing extreme values in these cases. Conversely, GBLUP performs slightly better in datasets like Groundnut, Indica, Japonica, Maize, and Wheat_6, although these differences are relatively minor. Overall, DL tends to excel in metrics related to correlation and extreme value identification, while GBLUP demonstrates slightly more robust performance in error minimization, making each model more suitable for specific predictive objectives. See Figure 6.

## Discussion

The comparative analysis of DL and GBLUP models provides valuable insights into their predictive performance across multiple metrics: (1) Box plot of the Pearson’s correlation (Cor) between observed and predicted values for each trait. (2) Box plot of the normalized root mean square error (NRMSE) between observed and predicted values for each trait, an (3) Box plot of the top 20% matching percentages (Matching20) for observed and predicted values.

Correlation, NRMSE, and Matching20. These findings underscore the relative strengths and limitations of each model, with implications for their application in diverse datasets and traits.

DL frequently achieved higher correlation scores, particularly for traits with complex relationships, as observed in GY within the EYT_11 and EYT_3 datasets. This suggests that DL’s architecture and non-linear modeling capabilities are well-suited for capturing intricate patterns in data. In contrast, GBLUP excelled for simpler traits, such as NPP in the Groundnut dataset, where the linear assumptions of the model align well with the underlying trait relationships. These findings are consistent with previous studies highlighting the flexibility of DL for complex traits (Zingaretti et al., 2020; Montesinos-López et al., 2021) and the reliability of GBLUP for more straightforward cases (Meuwissen et al., 2001).

An important strength of this study lies in the diversity of the datasets analyzed, which vary in crop species, trait types, and sample sizes. This allowed us to evaluate DL’s robustness across practical breeding scenarios. The consistent performance of DL, even in smaller datasets, suggests it may be a valuable tool for programs with limited training data, expanding the accessibility of advanced modeling approaches beyond large-scale breeding operations.

The 14 datasets analyzed in this study vary significantly in complexity, offering a robust test bed for comparing DL and GBLUP models. Dataset complexity stems from three main factors: trait architecture, sample size, and marker density. Traits like grain yield (GY), disease resistance (e.g., Septoria nodorum and spot blotch), and plant height reduction (PHR) are considered complex due to their polygenic nature and strong genotype-by-environment (G × E) interactions. In contrast, traits such as gel consistency (GC) and days to heading (DTHD) are simpler and mostly controlled by additive effects. Sample sizes ranged from small datasets like Groundnut (318 lines) and Indica (327 lines) to larger datasets such as Wheat_2 (1,403 lines) and Wheat_5 (1,398 lines), reflecting the variability in breeding program scale. Marker density also differed, with low-density datasets like EYT_1 to EYT_3 (2,038 SNPs) and high-density datasets like Wheat and Maize (over 78,000 and 54,000 SNPs, respectively). This combination of diverse traits, species, population sizes, and genomic resolutions enabled a comprehensive assessment of model performance across realistic breeding scenarios with varying levels of data complexity.

The near-identical predictive errors exhibited by both models, reflected in minimal differences in NRMSE, suggest comparable performance in terms of absolute prediction accuracy. This result indicates that while DL may capture complex patterns better, its advantage does not necessarily translate to reduced overall error. Although advanced models, such as deep learning (DL), have shown promise in leveraging complex patterns within genomic data, they do not always lead to substantial improvements in general error metrics when compared to traditional models like GBLUP in genomic prediction settings. This is likely because GBLUP, with its simplicity and reliance on additive genetic effects, often captures a significant portion of the genetic signal in well-structured datasets, making it challenging for more complex models to consistently outperform it without additional sources of information or better data quality (Crossa et al., 2017).

The finding that deep learning (DL) consistently outperformed GBLUP in the Matching20 scenario underscores the strength of DL in handling ranking tasks, which are critical for prioritizing individuals or genotypes in breeding programs. The superior ranking accuracy of DL suggests that it can more effectively capture complex, nonlinear interactions within genomic data, as well as subtle patterns that traditional models like GBLUP may overlook. This advantage becomes particularly valuable when the goal is not just overall prediction accuracy but also the accurate identification of the top-performing individuals or genotypes within a population.

From a practical standpoint, this result highlights DL’s potential to generate actionable insights for breeding and selection programs. For example, in scenarios where resource constraints limit the number of genotypes that can be advanced or tested in field trials, accurately identifying high-value candidates becomes essential. By leveraging DL’s superior ranking capabilities, breeders can focus their resources on individuals with the greatest potential to contribute to program goals, such as increased yield, stress tolerance, or disease resistance (Crossa et al., 2017).

Moreover, the practical implications of DL’s performance extend beyond simple rankings. The ability to reliably identify top performers also supports long-term strategic decisions, such as the design of crossing schemes or the development of elite lines, ultimately accelerating genetic gain. However, while these results are promising, it is important to consider that the benefits of DL may depend on factors such as the quality and quantity of the data, the complexity of the traits being studied, and the computational resources available for model training and implementation (Ma et al., 2018; Montesinos-López et al., 2021).

The theoretical development explains why DL models may be superior to GBLUP in certain genomic prediction scenarios largely revolves around their ability to model complex, nonlinear relationships and interactions among features (markers). GBLUP relies on a linear mixed model framework where relationships between predictors (genomic markers) and the target trait are assumed to be linear. While effective for traits predominantly governed by additive genetic effects, this assumption breaks down when epistasis (gene-by-gene interactions) plays a significant role or when marker effects exhibit non-additive relationships (dominance or epistatic effects).

DL, by contrast, employs neural network architectures capable of learning highly nonlinear and complex relationships among features without explicit feature engineering (LeCun et al., 2015). This makes DL particularly suitable for traits governed by epistatic or non-additive genetic effects (Montesinos-López et al., 2021).

Another critical distinction lies in scalability. While GBLUP is computationally efficient for moderate-sized datasets, it scales poorly with very large datasets. Conversely, DL models thrive as data size increases. Theoretically, the more data provided, the better DL models can generalize, given that they are less prone to underfitting in high-dimensional settings (Williams and Rasmussen, 2006).

Despite these theoretical advantages, DL does not universally outperform GBLUP in practice, particularly when: 1) the dataset is small, making DL prone to overfitting; 2) the genetic architecture of the trait is simple; 3) most variation is explained by additive effects, where GBLUP’s simplicity suffices (Crossa et al., 2017); or 4) interpretability of the model is a priority, as DL often acts as a “black box”. Additional theoretical justification is provided in Supplementary Appendix C, supported by existing research.

### DL and genotype × environment interaction

Several recent studies have explored deep learning frameworks that explicitly model genotype-by-environment interactions (G × E) and multi-trait architectures in genomic prediction. For example, Montesinos-López et al. (2018) proposed multi-trait, multi-environment deep learning (MTME-DL) models, extending the classical single-output multilayer perceptron to handle correlated outputs and environment-specific effects. More recently, multimodal deep learning (MMDL) frameworks (Montesinos-López et al., 2023) have demonstrated the capacity of DL models to integrate genomic, environmental, and phenotypic data through parallel network branches, capturing cross-modal interactions. Similarly, convolutional neural networks (CNNs) and recurrent neural networks (RNNs) have been applied to sequence data or longitudinal environmental information, offering a path to encode temporal or spatial G × E structures (Ma et al., 2018; Zingaretti et al., 2020). While such architectures are powerful, they also introduce increased complexity in hyperparameter tuning, data preprocessing, and model interpretation, which was beyond the scope of the present benchmarking study. Nonetheless, our work provides an essential baseline by comparing standard MLPDL and GBLUP under well-controlled conditions, and it can serve as a springboard for future research into integrated DL models better suited to the full spectrum of breeding program realities.

While hyperparameter tuning is critical for optimizing DL model performance, we acknowledge that this process can be time-consuming and computationally intensive, especially for large datasets. In our experience, using a fixed set of default hyperparameters based on previous genomic prediction studies can yield reasonable results. However, models tuned through Bayesian optimization consistently outperformed those using default settings. We recommend that, when possible, tuning should be applied, particularly for smaller or more complex datasets where predictive accuracy gains can be substantial. For practitioners with limited resources, a hybrid strategy that begins with default settings and selectively tunes key parameters may offer a practical compromise.

It is noteworthy that the 14 datasets analyzed are relatively small in terms of sample size. This aspect is particularly significant because it highlights the robustness of deep learning models in genomic prediction, even when working with limited data. Typically, deep learning models are known for requiring large volumes of data to achieve optimal performance due to their complex architectures and numerous parameters (LeCun et al., 2015; Goodfellow et al., 2016). However, our results provide empirical evidence that these models can still perform effectively in small sample contexts, a scenario common in genomic studies where collecting large datasets may be constrained by cost, time, or biological limitations.

The implications of these findings are very interesting. They suggest that DL methods can be a viable tool for genomic prediction even in resource-constrained settings. This expands the accessibility of cutting-edge predictive models to smaller research facilities and projects, ultimately accelerating discoveries in plant breeding, personalized medicine, and other genomics-driven fields. The ability of deep learning to derive meaningful insights from limited data underscores its potential to transform genomic research, offering a powerful approach to address complex biological questions with minimal data resources.

While our results demonstrate consistent advantages of DL over GBLUP in several scenarios, it is important to note that the 10-fold cross-validation strategy employed in this study, although standard in genomic prediction literature, was not repeated multiple times. We reported standard deviations across folds to partially capture variability in performance. However, future investigations with access to larger computational resources could explore a more exhaustive evaluation using a high number of repetitions (e.g., 100 or 1,000) to assess the full robustness of the observed trends in predictive ability across all models and datasets.

## Conclusion

The results highlight the nuanced differences between DL and GBLUP models across datasets and traits. DL often excelled in traits involving complex relationships (e.g., GY in EYT datasets), reflecting its ability to capture non-linear patterns. GBLUP, however, showed competitive performance for traits with simpler structures dominated by additive genetics effects (e.g., NPP and PYPP in the Groundnut dataset, and GC, GY and PH in Japonica dataset). Standard deviations indicate consistency across folds, with most metrics showing low variability. This comprehensive comparison underscores the complementary strengths of both models and the need for trait-specific and metrics prioritized model selection in predictive analyses, as neither DL nor GBLUP universally outperformed the other.

## Data Availability

The original contributions presented in the study are included in the article/Supplementary Material, further inquiries can be directed to the corresponding authors.
