# Supplementary material for: Artificial intelligence meets genomic selection: comparing deep learning and GBLUP across diverse plant datasets
Source: Front Genet. 2025 Apr 29;16:1568705. doi: 10.3389/fgene.2025.1568705 (PMC12069277; doi:10.3389/fgene.2025.1568705)
Supplement: Supplementary file 1 [file DataSheet1.docx]

**Appendix A**

**Table A1.** Prediction performance of deep learning (DL) and GBLUP models in each of the 14 datasets and traits explored. The mean values across the folds for each of the metrics used were reported: Pearson correlation (Cor), normalized mean squared error (NRMSE), and the matching values in the top 20% (Matching20).Traits are: Pyrenophora tritici-repentis (PTR), spot blocht (SB), and Septoria nodorum (SN), days to heading (DTHD), days to maturity (DTMT), Grain Yield (GY, Y), plant height (HEIGHT), Number of Pods per Plant (NPP), Pod Yield per Plant (PYPP), Seed Yield per Plant (SYPP), Yield per Hectare (YPH), Gel Consistency (GC), Grain yield (GY), Plant height (PH), Plant Height Reduction (PHR).

| **Model** | **Dataset** | **Trait** | **Cor (SD)** | **NRMSE (SD)** | **Matching20 (SD)** |
| --- | --- | --- | --- | --- | --- |
| DL | Disease | PTR | 0.213 (0.08) | 0.420 (0.04) | 0.311 (0.11) |
| GBLUP | Disease | PTR | 0.193 (0.10) | 0.423 (0.03) | 0.222 (0.10) |
| DL | Disease | SB | 0.244 (0.12) | 0.378 (0.03) | 0.344 (0.09) |
| GBLUP | Disease | SB | 0.260 (0.12) | 0.375 (0.03) | 0.311 (0.14) |
| DL | Disease | SN | 0.138 (0.18) | 0.466 (0.03) | 0.244 (0.08) |
| GBLUP | Disease | SN | 0.125 (0.06) | 0.469 (0.03) | 0.255 (0.10) |
| DL | EYT_1 | DTHD | 0.554 (0.09) | 0.053 (0) | 0.427 (0.11) |
| GBLUP | EYT_1 | DTHD | 0.523 (0.06) | 0.054 (0) | 0.416 (0.09) |
| DL | EYT_1 | DTMT | 0.525 (0.10) | 0.034 (0) | 0.416 (0.09) |
| GBLUP | EYT_1 | DTMT | 0.503 (0.08) | 0.035 (0) | 0.416 (0.07) |
| DL | EYT_1 | GY | 0.479 (0.09) | 0.053 (0) | 0.482 (0.12) |
| GBLUP | EYT_1 | GY | 0.481 (0.09) | 0.053 (0) | 0.418 (0.15) |
| DL | EYT_1 | Height | 0.444 (0.1) | 0.035 (0) | 0.420 (0.14) |
| GBLUP | EYT_1 | Height | 0.425 (0.09) | 0.036 (0) | 0.370 (0.11) |
| DL | EYT_2 | DTHD | 0.523 (0.11) | 0.042 (0) | 0.412 (0.08) |
| GBLUP | EYT_2 | DTHD | 0.479 (0.09) | 0.044 (0) | 0.412 (0.08) |
| DL | EYT_2 | DTMT | 0.593 (0.07) | 0.024 (0) | 0.512 (0.08) |
| GBLUP | EYT_2 | DTMT | 0.563 (0.08) | 0.024 (0) | 0.512 (0.10) |
| DL | EYT_2 | GY | 0.616 (0.08) | 0.050 (0) | 0.556 (0.11) |
| GBLUP | EYT_2 | GY | 0.597 (0.08) | 0.051 (0) | 0.487 (0.10) |
| DL | EYT_2 | Height | 0.516 (0.05) | 0.032 (0) | 0.493 (0.08) |
| GBLUP | EYT_2 | Height | 0.499 (0.08) | 0.032 (0) | 0.431 (0.09) |
| DL | EYT_3 | DTHD | 0.530 (0.08) | 0.034 (0) | 0.474 (0.07) |
| GBLUP | EYT_3 | DTHD | 0.504 (0.08) | 0.035 (0) | 0.423 (0.08) |
| DL | EYT_3 | DTMT | 0.564 (0.04) | 0.019 (0) | 0.551 (0.07) |
| GBLUP | EYT_3 | DTMT | 0.512 (0.06) | 0.020 (0) | 0.463 (0.07) |
| DL | EYT_3 | GY | 0.571 (0.05) | 0.049 (0) | 0.532 (0.08) |
| GBLUP | EYT_3 | GY | 0.527 (0.04) | 0.050 (0) | 0.475 (0.07) |
| DL | EYT_3 | Height | 0.568 (0.04) | 0.030 (0) | 0.484 (0.07) |
| GBLUP | EYT_3 | Height | 0.524 (0.04) | 0.031 (0) | 0.488 (0.08) |
| DL | Groundnut | NPP | 0.648 (0.07) | 0.206 (0.02) | 0.469 (0.10) |
| GBLUP | Groundnut | NPP | 0.670 (0.08) | 0.200 (0.02) | 0.483 (0.08) |
| DL | Groundnut | PYPP | 0.594 (0.12) | 0.200 (0.03) | 0.523 (0.20) |
| GBLUP | Groundnut | PYPP | 0.623 (0.11) | 0.194 (0.03) | 0.538 (0.19) |
| DL | Groundnut | SYPP | 0.587 (0.11) | 0.221 (0.03) | 0.452 (0.13) |
| GBLUP | Groundnut | SYPP | 0.603 (0.11) | 0.218 (0.03) | 0.495 (0.14) |
| DL | Groundnut | YPH | 0.653 (0.13) | 0.257 (0.03) | 0.583 (0.16) |
| GBLUP | Groundnut | YPH | 0.643 (0.16) | 0.257 (0.04) | 0.569 (0.15) |
| DL | Indica | GC | 0.419 (0.21) | 0.431 (0.09) | 0.442 (0.17) |
| GBLUP | Indica | GC | 0.403 (0.20) | 0.435 (0.1) | 0.442 (0.18) |
| DL | Indica | GY | 0.616 (0.10) | 0.056 (0) | 0.300 (0.12) |
| GBLUP | Indica | GY | 0.628 (0.09) | 0.055 (0) | 0.371 (0.13) |
| DL | Indica | PH | 0.541 (0.12) | 0.042 (0) | 0.500 (0.13) |
| GBLUP | Indica | PH | 0.537 (0.14) | 0.042 (0) | 0.557 (0.14) |
| DL | Indica | PHR | 0.378 (0.24) | 0.034 (0) | 0.385 (0.24) |
| GBLUP | Indica | PHR | 0.431 (0.25) | 0.033 (0) | 0.400 (0.21) |
| DL | Japonica | GC | 0.550 (0.21) | 0.252 (0.02) | 0.600 (0.13) |
| GBLUP | Japonica | GC | 0.563 (0.14) | 0.250 (0.01) | 0.542 (0.11) |
| DL | Japonica | GY | 0.505 (0.11) | 0.067 (0.01) | 0.514 (0.13) |
| GBLUP | Japonica | GY | 0.571 (0.11) | 0.063 (0.01) | 0.571 (0.16) |
| DL | Japonica | PH | 0.634 (0.07) | 0.042 (0.01) | 0.485 (0.16) |
| GBLUP | Japonica | PH | 0.608 (0.06) | 0.043 (0.01) | 0.500 (0.13) |
| DL | Japonica | PHR | 0.536 (0.11) | 0.030 (0) | 0.471 (0.09) |
| GBLUP | Japonica | PHR | 0.545 (0.10) | 0.030 (0) | 0.400 (0.14) |
| DL | Maize | Y | 0.43 (0.08) | 124.703 (385.36) | 0.433 (0.10) |
| GBLUP | Maize | Y | 0.435 (0.06) | 124.323 (384.01) | 0.446 (0.11) |
| DL | Wheat_1 | GY | 0.549 (0.04) | 0.047 (0) | 0.419 (0.08) |
| GBLUP | Wheat_1 | GY | 0.504 (0.05) | 0.048 (0) | 0.407 (0.05) |
| DL | Wheat_2 | GY | 0.413 (0.08) | 0.041 (0) | 0.407 (0.05) |
| GBLUP | Wheat_2 | GY | 0.394 (0.08) | 0.041 (0) | 0.403 (0.08) |
| DL | Wheat_3 | GY | 0.475 (0.04) | 0.041 (0) | 0.438 (0.06) |
| GBLUP | Wheat_3 | GY | 0.478 (0.05) | 0.041 (0) | 0.415 (0.04) |
| DL | Wheat_4 | GY | 0.403 (0.06) | 0.042 (0) | 0.475 (0.04) |
| GBLUP | Wheat_4 | GY | 0.376 (0.02) | 0.043 (0) | 0.442 (0.05) |
| DL | Wheat_5 | GY | 0.454 (0.08) | 0.037 (0) | 0.432 (0.07) |
| GBLUP | Wheat_5 | GY | 0.448 (0.05) | 0.037 (0) | 0.417 (0.05) |
| DL | Wheat_6 | GY | 0.558 (0.04) | 0.040 (0) | 0.519 (0.08) |
| GBLUP | Wheat_6 | GY | 0.548 (0.04) | 0.040 (0) | 0.550 (0.08) |

**Table A2.** Average prediction performance across folds and traits for deep learning (DL) and GBLUP models in each of the 14 datasets explored, evaluated using three metrics: Pearson correlation (Cor), normalized mean squared error (NRMSE), and matching values in the top 20% (Matching20).

| **Model** | **Dataset** | **Cor (SD)** | **NRMSE (SD)** | **Matching20 (SD)** | |
| --- | --- | --- | --- | --- | --- |
| DL | Disease | 0.199 (0.054) | 0.421 (0.044) | 0.300 (0.050) |  |
| GBLUP | Disease | 0.193 (0.067) | 0.423 (0.046) | 0.262 (0.044) |  |
| DL | EYT_1 | 0.501 (0.048) | 0.044 (0.010) | 0.436 (0.030) |  |
| GBLUP | EYT_1 | 0.483 (0.042) | 0.044 (0.010) | 0.405 (0.023) |  |
| DL | EYT_2 | 0.562 (0.05) | 0.037 (0.011) | 0.493 (0.060) |  |
| GBLUP | EYT_2 | 0.534 (0.054) | 0.038 (0.011) | 0.460 (0.046) |  |
| DL | EYT_3 | 0.558 (0.019) | 0.033 (0.012) | 0.510 (0.037) |  |
| GBLUP | EYT_3 | 0.517 (0.01) | 0.034 (0.012) | 0.462 (0.028) |  |
| DL | Groundnut | 0.620 (0.034) | 0.221 (0.025) | 0.507 (0.059) |  |
| GBLUP | Groundnut | 0.635 (0.028) | 0.217 (0.028) | 0.521 (0.039) |  |
| DL | Indica | 0.489 (0.109) | 0.141 (0.193) | 0.407 (0.085) |  |
| GBLUP | Indica | 0.500 (0.103) | 0.141 (0.195) | 0.442 (0.081) |  |
| DL | Japonica | 0.556 (0.054) | 0.098 (0.103) | 0.517 (0.057) |  |
| GBLUP | Japonica | 0.572 (0.026) | 0.096 (0.103) | 0.503 (0.075) |  |
| DL | Maize | 0.430 (0.000) | 124.703 (0) | 0.433 (0) |  |
| GBLUP | Maize | 0.435 (0) | 124.323 (0) | 0.446 (0) |  |
| DL | Wheat_1 | 0.549 (0) | 0.047 (0) | 0.419 (0) |  |
| GBLUP | Wheat_1 | 0.504 (0) | 0.048 (0) | 0.407 (0) |  |
| DL | Wheat_2 | 0.413 (0) | 0.041 (0) | 0.407 (0) |  |
| GBLUP | Wheat_2 | 0.394 (0) | 0.041 (0) | 0.403 (0) |  |
| DL | Wheat_3 | 0.475 (0) | 0.041 (0) | 0.438 (0) |  |
| GBLUP | Wheat_3 | 0.478 (0) | 0.041 (0) | 0.415 (0) |  |
| DL | Wheat_4 | 0.403 (0) | 0.042 (0) | 0.475 (0) |  |
| GBLUP | Wheat_4 | 0.376 (0) | 0.043 (0) | 0.442 (0) |  |
| DL | Wheat_5 | 0.454 (0) | 0.037 (0) | 0.432 (0) |  |
| GBLUP | Wheat_5 | 0.448 (0) | 0.037 (0) | 0.417 (0) |  |
| DL | Wheat_6 | 0.558 (0) | 0.040 (0) | 0.519 (0) |  |
| GBLUP | Wheat_6 | 0.548 (0) | 0.040 (0) | 0.550 (0) |  |

**Appendix B**

**
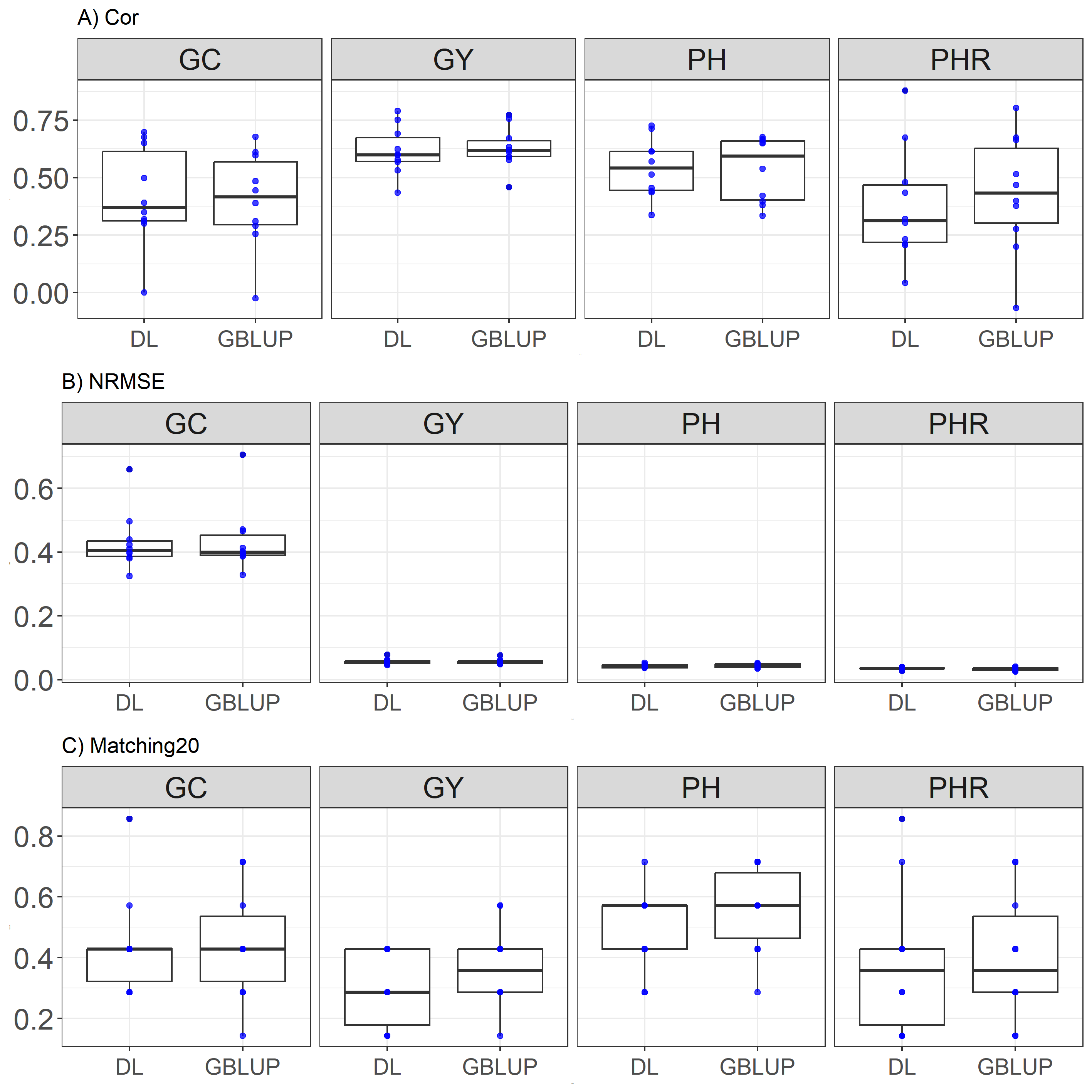
Figure B1**. Box plots depicting the performance of DL and GBLUP models across ten-fold cross-validation for *Indica* data in each trait, Gel Consistency (GC), Grain yield (GY), Plant height (PH), Plant Height Reduction (PHR*)*. A) Box plot of the Pearson's correlation (Cor) between observed and predicted values for each of the three traits across ten-fold cross-validation. B) Box plot of the normalized root mean square error (NRMSE) between observed and predicted values for each trait. C) Box plot of the top 20% matching percentages (Matching20) for observed and predicted values.

**
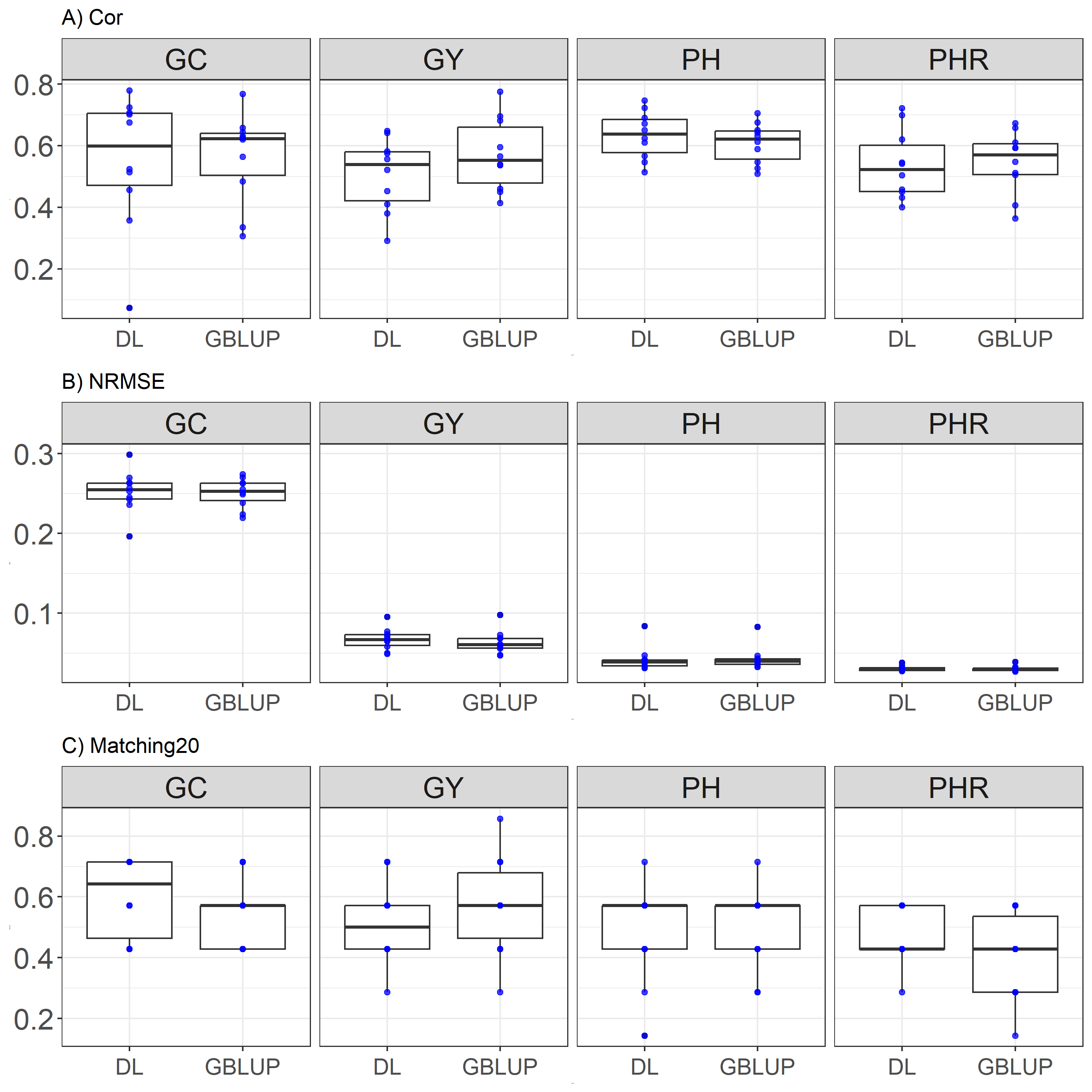
Figure B2**. Box plots depicting the performance of DL and GBLUP models across ten-fold cross-validation for *Japonica* data in each trait, Gel Consistency (GC), Grain yield (GY), Plant height (PH), Plant Height Reduction (PHR). A) Box plot of the Pearson's correlation (Cor) between observed and predicted values for each of the three traits across ten-fold cross-validation. B) Box plot of the normalized root mean square error (NRMSE) between observed and predicted values for each trait. C) Box plot of the top 20% matching percentages (Matching20) for observed and predicted values.


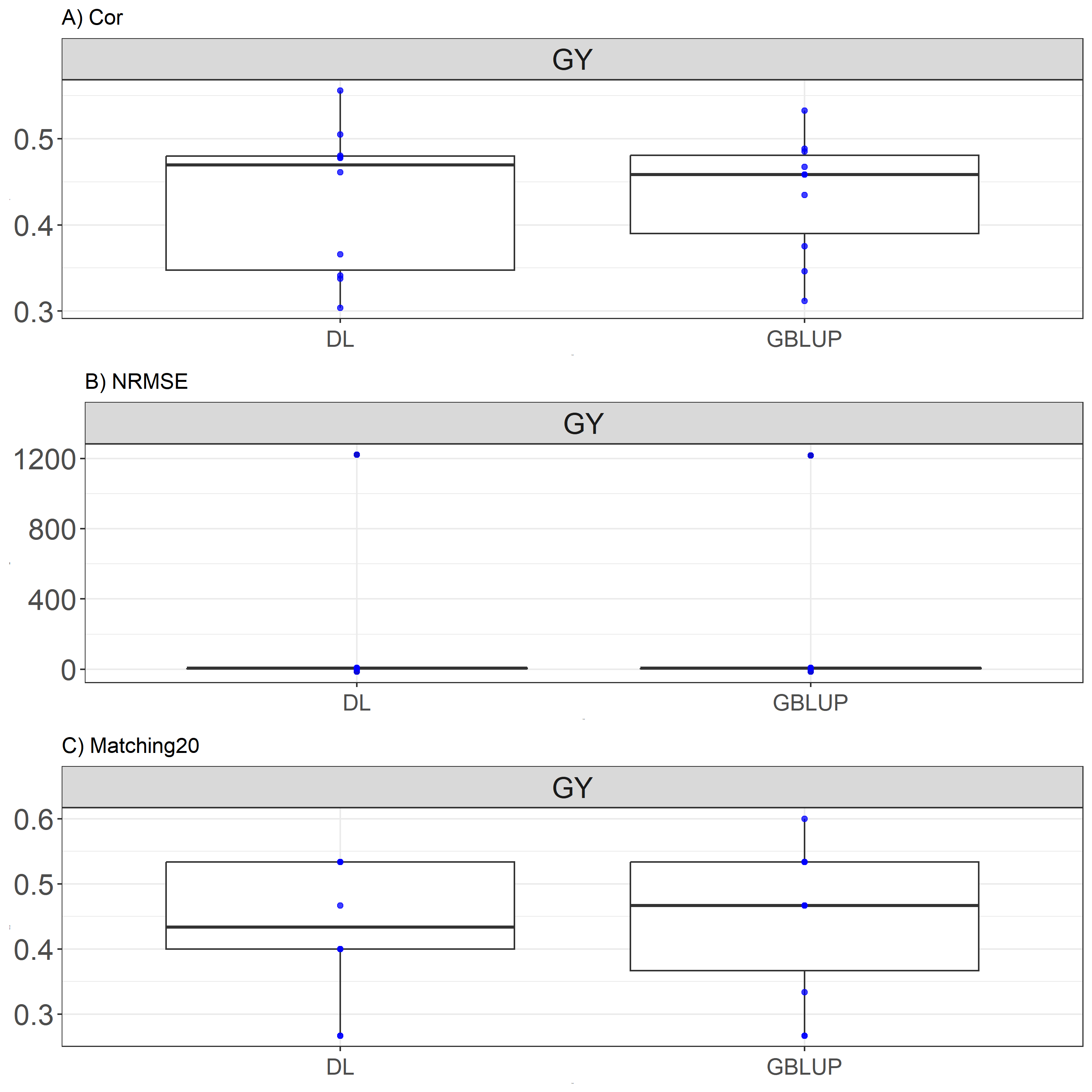
**Figure B3**. Box plots for Maize dataset showing the performance of the DL and GBLUP methods through ten-fold cross-validation for the unique trait grain yield (GY). A) Box plot of the performance using Pearson’s correlation (Cor) between observed and predicted values. B) Box plot of the performance using the Normalized Root Mean Squared Error (NRMSE) between observed and predicted values through ten-fold cross-validation. C) Box plot of the performance using the matching values in the top 20% (Matching20) between observed and predicted values.

**
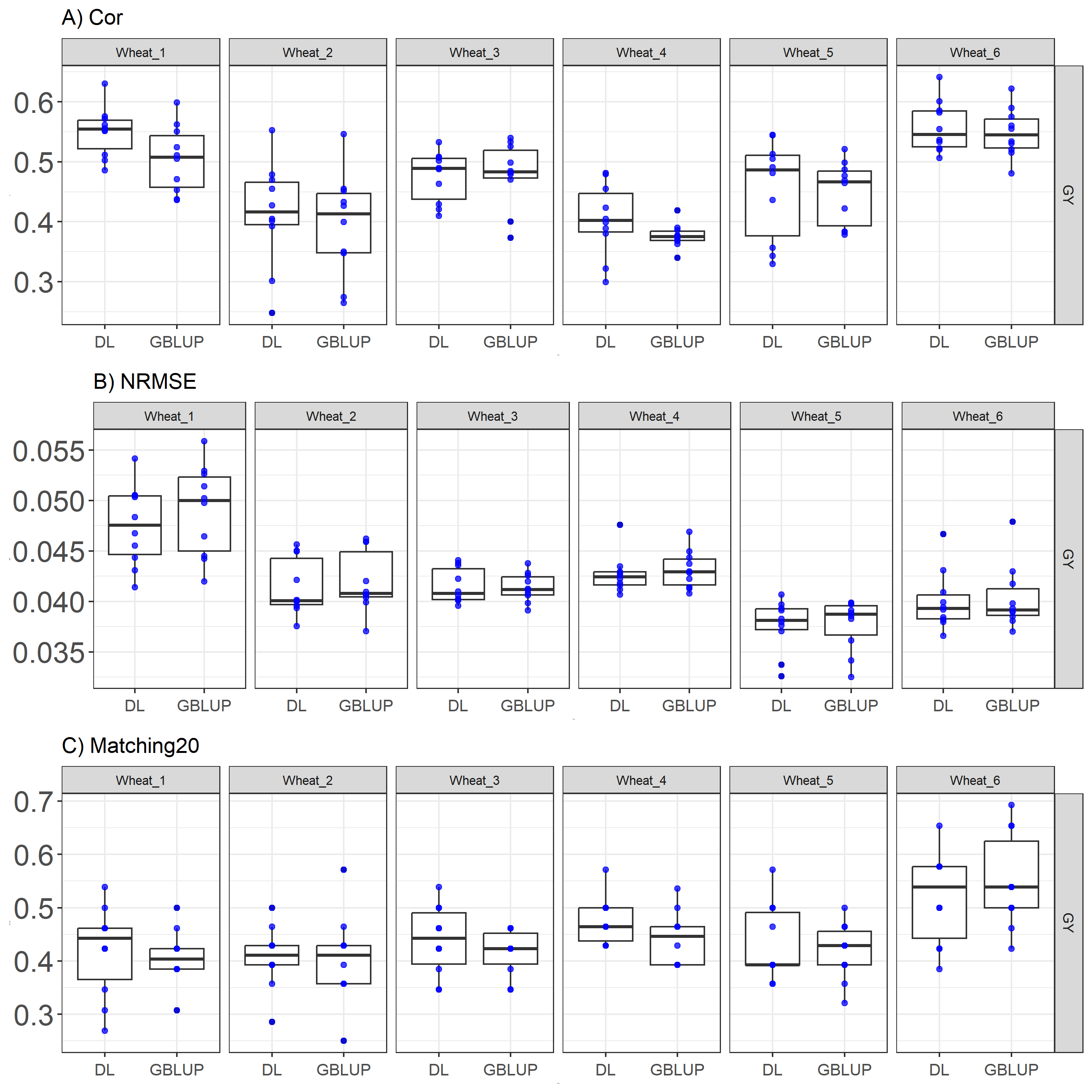
Figure B4**. Box plots for Wheat_1 to Wheat_6 dataset showing the performance of the DL and GBLUP methods through ten-fold cross-validation for the unique trait grain yield (GY). A) Box plot of the performance using Pearson’s correlation (Cor) between observed and predicted values. B) Box plot of the performance using the Normalized Root Mean Squared Error (NRMSE) between observed and predicted values through ten-fold cross-validation. C) Box plot of the performance using the matching values in the top 20% (Matching20) between observed and predicted values.

**Appendix C**

**Handling High-Dimensional and Complex Data**

GBLUP struggles with high-dimensional genomic data when the number of markers greatly exceeds the number of samples (p≫n) unless sparsity is introduced. DL, on the other hand, can efficiently process large-scale datasets by:

- Reducing dimensionality through learned representations (e.g., embeddings).
- Exploiting complex patterns in high-dimensional spaces through deep network layers.

In genomic prediction, DL models have been shown to learn hierarchical relationships between markers, capturing both local and global patterns (Ma et al., 2018).

**Feature Interactions and Representation Learning**

GBLUP assumes pre-specified kernel functions (e.g., the genomic relationship matrix), which inherently limit its ability to adaptively capture interactions among features. DL excels in this aspect:

- Neural networks dynamically learn feature representations, allowing them to adaptively model interactions (LeCun et al., 2015).
- This flexibility helps DL uncover latent genetic structures and interactions missed by GBLUP.

**Multi-Modal and Contextual Information Integration**

DL frameworks are theoretically better suited for integrating diverse data types (e.g., genomic, environmental, phenotypic) due to their modularity. For example:

- Convolutional Neural Networks (CNNs) capture spatial relationships in SNP data.
- Recurrent Neural Networks (RNNs) capture temporal or sequential patterns, such as gene expression changes over time.

By contrast, GBLUP lacks inherent mechanisms to fuse diverse data types efficiently (Crossa et al., 2017).

**References for Further Reading**

LeCun, Y., Bengio, Y., & Hinton, G. (2015). Deep learning. *Nature*, 521(7553), 436–444.

Ma, W., Qiu, Z., Song, J., Li, J., Cheng, Q., Zhai, J., ... & Zhang, W. (2018). A deep learning approach for predicting phenotypes from genotypes. *Frontiers in Genetics*, 9, 237.

Crossa, J., Pérez-Rodríguez, P., Cuevas, J., Montesinos-López, O., Jarquín, D., de los Campos, G., ... & Montesinos-López, A. (2017). Genomic selection in plant breeding: Methods, models, and perspectives. *Trends in Plant Science*, 22(11), 961–975.

Montesinos-López, O. A., Montesinos-López, A., Crossa, J., de los Campos, G., Alvarado, G., Suchan, J., & Burgueno, J. (2021). A review of deep learning applications for genomic selection. *BMC Genomics*, 22(1), 19.

Montesinos-López, O. A., Montesinos-López, A., Crossa, J., Gianola, D., Hernández-Suárez, C. M., and Martín-Vallejo, J. (2018). Multi-trait, multi-environment deep learning modeling for genomic-enabled prediction of plant traits. G3: Genes, Genomes, Genetics, 8(12), 3829-3840. https://doi.org/10.1534/g3.118.200740

Montesinos-López, O. A., Montesinos-López, A., Pérez-Rodríguez, P., Barrón-López, J. A., Martini, J. W., Fajardo-Flores, S. B., and Crossa, J. (2021). A review of deep learning applications for genomic selection. BMC Genomics, 22, 1–23. https://doi.org/10.1186/s12864-020-07319-x

Montesinos-López, A., Rivera, C., Pinto, F., Piñera, F., Gonzalez, D., Reynolds, M., and Crossa, J. (2023). Multimodal deep learning methods enhance genomic prediction of wheat breeding. G3: Genes, Genomes, Genetics, 13(5), jkad045. <https://doi.org/10.1093/g3journal/jkad045>

Rasmussen, C. E., & Williams, C. K. I. (2006). *Gaussian Processes for Machine Learning*. MIT Press, Boston, MA.
